# Prevalence and genotypic characterization of *Giardia duodenalis* isolates from asymptomatic school-going children in Lusaka, Zambia

**DOI:** 10.1016/j.fawpar.2020.e00072

**Published:** 2020-02-21

**Authors:** Shadreck J. Tembo, Mable M. Mutengo, Lungowe Sitali, Katendi Changula, Ayato Takada, Aaron S. Mweene, Edgar Simulundu, Simbarashe Chitanga

**Affiliations:** a Department of Biomedical Sciences, School of Health Sciences, University of Zambia, Zambia; b Parasitology Unit, Department of Pathology and Microbiology, University Teaching Hospital, Ministry of Health, Zambia; c Department of Paraclinical Studies, School of Veterinary Medicine, University of Zambia, Zambia; d Department of Disease Control, School of Veterinary Medicine, University of Zambia, Zambia; e Division of Global Epidemiology, Hokkaido University Research Center for Zoonosis Control, Sapporo, Japan; f Africa Center of Excellence for Infectious Diseases of Humans and Animals, 10101 Lusaka, Zambia

**Keywords:** *Giardia duodenalis*, Giardiasis, Genotyping, Glutamate dehydrogenase, Phylogenetic analysis, Zambia

## Abstract

*Giardia duodenalis* is one of the most common causes of diarrhea in humans with about 250–300 million cases per year. It is considered to be a species complex comprising of eight genetic assemblages (A to H), with assemblages A and B being the major causes of human infections. In this study we carried out genotypic characterization of *G. duodenalis* isolates detected in asymptomatic school-going children aged 3–16 years. Between May and September 2017, a total of 329 fecal samples were collected from school-going children from Chawama compound of Lusaka City and were screened for *Giardia* by microscopic examination. All microscopically positive fecal samples were analyzed by semi-nested polymerase chain reaction (PCR) targeting the glutamate dehydrogenase (*gdh*) gene. Genotyping of amplified PCR products was conducted by restriction fragment length polymorphism (RFLP) and DNA sequence analysis. Microscopically, *Giardia* was found in 10% (33/329) of fecal samples. The PCR-RFLP analysis of the *gdh* gene revealed assemblages A and B in 27.3% (9/33) and 72.7% (24/33), respectively. Furthermore, analysis with restriction enzymes identified sub-assemblages AII (27.3%, 9/33), BIII (12.1%, 4/33), BIV (51.5%, 17/33) and mixed infections of BIII and BIV (9.1%, 3/33). Phylogenetic analysis showed the clustering of 27.6% (8/29) and 72.4% (21/29) of Zambian *Giardia gdh* gene sequences into assemblages A and B, respectively. This study has revealed the presence of both assemblage A and B and that spread of *G. duodenalis* in school-going children appears to be mostly through anthroponotic transmission. To our knowledge, this is the first report of genotypic characterization of *G. duodenalis* identified in Zambia.

## Highlights

• *Giardia duodenalis* infection observed in asymptomatic children
• First report of genotypes circulating in Zambia
• Presence of sub-assemblages AII, BIII and BIV
• Co-infection with sub-assemblages BIII and BIV reported

## Graphical abstract

**Figure:**
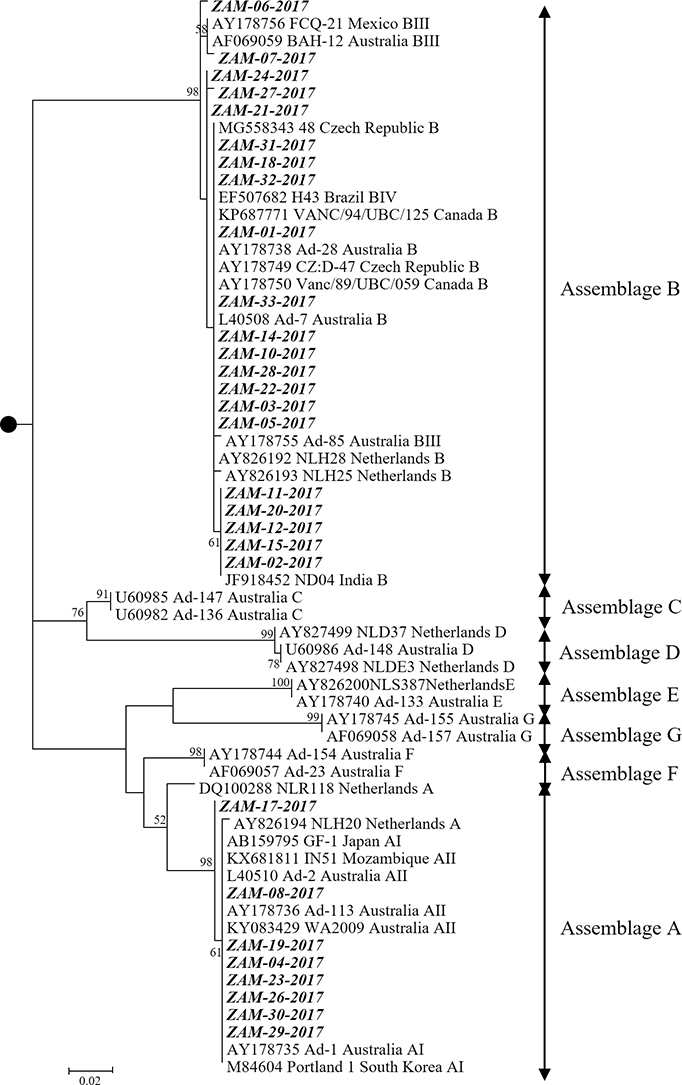

## 1 Introduction

*Giardia duodenalis* is a protozoan parasite which is responsible for 250–300 million symptomatic human infections annually, with less developed communities being the most afflicted (Einarsson et al., 2016). The infection can manifest as acute diarrhea, which may become chronic if not treated, with majority of infections being asymptomatic (Certad et al., 2017). Chronic infection is associated with malabsorption which can lead to subsequent weight loss and wasting in children (Squire and Ryan, 2017). *G. duodenalis* is also reported to be associated with cognitive impairment in school-age children (Berkman et al., 2002). Transmission of the pathogen occurs either directly (fecal-oral route) or indirectly (through contaminated food and water), with the indirect route being the major route of transmission (Thompson, 2000).

*G. duodenalis* is considered to be a species complex, with eight genetic assemblages (A-H), whose host range varies widely (A and B – Humans and other mammals, C and D – Canids, E – Hooved animals, F – Cats, G – Rats and H – Marine mammals) (Minetti et al., 2016). However, there have been recent reports of humans being infected with assemblages E and F (Abdel-Moein and Saeed, 2016; Fantinatti et al., 2016; Gelanew et al., 2007; Zahedi et al., 2017). Assemblages A and B are further divided into sub-assemblages (AI, AII, AIII, AIV, BI, BII, BIII and BIV) (Monis et al., 2003). *G. duodenalis* assemblages can be distinguished based on single nucleotide polymorphism and/or genotypic analysis of the *ssu – rRNA*beta, (β)-giardin (*bg*), triose phosphate isomerase (*tpi*) and glutamate dehydrogenase (*gdh*) genes (Caccio and Ryan, 2008).

In Africa, the reported prevalence of *Giardia* infection varies widely possibly due to use of different diagnostic techniques, with most studies being based on microscopy and serology (Squire and Ryan, 2017). A few molecular studies in Africa have revealed the presence of assemblages A, B, C, E and F in the human population (Squire and Ryan, 2017). Characterization to sub-assemblage level has shown the presence of sub-assemblages AI, AII, BIII, BIV as well as some novel sub-assemblages (Squire and Ryan, 2017). Whilst the occurrence of giardiasis in children has been reported in Zambia (Graczyk et al., 2005; Siwila et al., 2010, Siwila et al., 2011), there has been no information on the genotypes present in the country. The purpose of this study was to determine the prevalence of *Giardia* infection in asymptomatic children in Lusaka as well as to genetically characterize the isolates infecting these children. Knowledge of the infective genotypes would lead to better understanding of the epidemiology of the disease and assist in designing appropriate control strategies.

## 2 Materials and methods

### 2.1 Fecal sample collection and microscopic examination

A cross-sectional study was conducted between May and September 2017, which is the dry season when the area is more accessible and before the beginning of national Grade seven (7) examinations, during which a total of 329 asymptomatic school-going children (146 boys and 183 girls) were sampled from two public (123) and two community (206) schools in Chawama compound of Lusaka District in Zambia. Only children who had not had diarrhea in the month preceding the date of sample collection were considered in this study, whilst those who had at least one bout of diarrhea in the same period were excluded from the study. Chawama is a high-density settlement within the City of Lusaka, whose origin is a result of unplanned settlement which was eventually officially recognized as a squatter settlement. Due to its unplanned settlement status, Chawama is characterized by inadequate sanitation and erratic water supply, with most households lacking adequate income (Ndhlema, 2000).

From each child, one fecal sample was collected in a 50 mL screw-capped container and processed by the formalin/ethyl acetate concentration method. The collected fecal samples were kept at 4 °C without preservation until processing and examination in the laboratory. Fecal consistency was noted as watery, soft, loose, semi-formed or formed. The fecal samples were examined for parasitic infection using wet smears stained with Lugol's iodine after being processed by the formal ether concentration method (Garcia, 2007). The intensity of infection was estimated as the average number of cysts count per high power field (HPF/ X40) of light microscope. The grading of *Giardia* infection was determined as described by Almeida et al. (2006). In brief, those with 1–2 cysts were recorded as 1+ (very low), 3–10 cysts recorded as 2+ (low), 11–30 cysts recorded as 3+ (medium or moderate), >30 cysts recorded as 4+ (high).

### 2.2 DNA extraction

All fecal samples that were *Giardia* positive by microscopic examination were stored at 2 to 8 °C prior to DNA extraction. Extraction of genomic DNA was performed directly from all positive stool samples using Fecal DNA MiniPrep (Zymo Research, USA) kit in accordance with the manufacturer's protocol. The extracted DNA samples were stored at −20 °C for PCR assays and other downward applications.

### 2.3 PCR amplification

Amplification of the 432 base pairs (bp) region of the *gdh* gene was carried out in a semi-nested PCR assay using primers and specific conditions described by Read et al. (2004). Briefly, in the first reaction, amplification was performed using the primer pair GDHeF: 5′-TCA ACG TYA AYC GYC GYT TCC GT-3′ and GDHiR: 5′-GTT RTC CTT GCA CAT CTC C-3′. The PCR product of this reaction was used in the second reaction with the primer set GDHiF: 5′-CAG TAC AAC TCY GCT CTC GG-3′ and GDHiR: 5′-GTT RTC CTT GCA CAT CTC C-3′. After electrophoresis, the final PCR product was visualized under ultraviolet (UV) light on 1.5% agarose gel stained with ethidium bromide.

### 2.4 PCR-RFLP analysis

The RFLP analysis was performed and interpreted as described by Read et al. (2004). Ten microliters (10 μL) of PCR product was added to 1 X enzyme buffer and 2 μL (10 U/μL) BspLI (NIaIV) or 2 μL (10 U / μL) *Rsa*I enzymes (New England Biolabs, USA) to make final volumes of 30 μL and 25 μL, respectively. The reaction mixture was incubated for a period of 16 h at 37 °C. The *Nla*IV enzyme digestion was used to distinguish assemblages A, B, C, D and sub-assemblages AI and AII. RsaI enzyme digestion was used to distinguish sub-assemblages BIII and BIV. Restriction fragments were separated on 3% agarose gel stained with ethidium bromide and visualized under UV light.

### 2.5 Sequence and phylogenetic analysis

Purification of PCR products, sequencing, sequence assembly and editing was conducted as previously described (Simulundu et al., 2018). The primer pair GDHiF/GDHiR was used for sequencing. The sequences generated in this study were deposited in GenBank (LC430549 – LC430577). Phylogenetic analysis was performed in MEGA V6.06 software using the Maximum Likelihood method and the Tamura-Nei evolutionary model (Tamura and Nei, 1993; Tamura et al., 2013). Model selection was conducted using MEGA V6.06 software (Tamura et al., 2013). Phylogenetic tree topological reliability was determined using the bootstrap method, with 1000 replicates.

### 2.6 Data analysis

Demographic data (sex, age, type of school) and presence of pathogen after stool microscopic examination was collected and entered into an Excel data spreadsheet for each child. The age of children ranged from 3 years to 16 years. Age was categorized into three groups of 3–6 years old, 7–9 years old and 10–16 years old, in line with the average age of children in pre-school, lower primary and upper primary grades, respectively. Prevalence was presented as percentage for all children as well as for the different categories (age, sex, type of school). Association of *Giardia* infection as a dependent variable with demographic factors as independent variables was assessed by Pearson's Chi Square test or Fischer's exact test were the number of outcomes was less than five. A *P* value of <0.05 (*P* < 0.05) was considered as the level of statistical significance for all tests. Data analysis was performed using Statistix version 9 (Analytical software).

### 2.7 Ethics statement

Ethical approval was granted by the University of Zambia Biomedical Research Ethics Committee (Reference no: 007-06-16). Fecal samples were collected only after written informed consent was obtained from parents and legal guardians for children to participate in the study. Permission to conduct the study from the various schools was obtained from the Lusaka District Education Board Secretary, Ministry of General Education in Zambia and Head teachers of the respective schools.

## 3 Results

### 3.1 Microscopic examination and genotyping of Giardia isolates by PCR-RFLP analysis

*Giardia* isolates were detected microscopically in 10.0% (33/329) of fecal samples collected from asymptomatic school-going children. Upon grading of infection level, the majority of the children (51.5%; 17/33) were found to have high infection levels (4+), with 21.2% (7/33), 15.2% (5/33) and 12.1% (4/33) having moderate (3+), low (2+) and very low (1+) infection levels, respectively. The prevalence amongst girls and boys was 12% (22/183) and 7.5% (11/146), respectively. By age group, the prevalence amongst the 3–6 year olds, 7–9 year olds and 10–16 year olds was 5.6%, 9.5% and 11.2%, respectively. The differences in prevalence between sexes and age group were not statistically significant (*P* > 0.05). However, statistically significant (*P* < 0.05) difference was observed in prevalence of infection between children attending public schools (15.4%; 19/123) and those attending community schools (6.8%; 14/206). Also, no statistically significant association was observed between *Giardia* assemblage and either sex or age group (P > 0.05).

The 432 bp fragment of the *gdh* gene was successfully amplified in all the 33 DNA samples (Table 1). The RFLP analysis of the *gdh* gene revealed assemblage A (27.3%, 9/33) and B (72.7%, 24/33) (Table 1). Furthermore, analysis with restriction enzymes identified sub-assemblages AII (27.3%, 9/33), BIII (12.1%, 4/33), BIV, (51.5%, 17/33) and mixed infections of BIII and BIV (9.1%, 3/33) (Table 1).

**Table 1:** Genotyping by PCR-RFLP and Phylogenetic analysis of *gdh* gene of *G. duodenalis* detected in school-going children in Zambia.

Isolate name	PCR-RFLP genotype		Phylogenetic clustering (assemblage)	GenBank accession no.
	Assemblage	Sub-assemblage		
ZAM-01-2017	B	BIV	B	LC430549
ZAM-02-2017	B	BIV	B	LC430550
ZAM-03-2017	B	BIV	B	LC430551
ZAM-04-2017	A	AII	A	LC430552
ZAM-05-2017	B	BIII	B	LC430553
ZAM-06-2017	B	BIV	B	LC430554
ZAM-07-2017	B	BIV	B	LC430555
ZAM-08-2017	A	AII	A	LC430556
ZAM-09-2017	B	BIII/BIV	NS	NS
ZAM-10-2017	B	BIV	B	LC430557
ZAM-11-2017	B	BIV	B	LC430558
ZAM-12-2017	B	BIV	B	LC430559
ZAM-13-2017	A	AII	NS	NS
ZAM-14-2017	B	BIII	B	LC430560
ZAM-15-2017	B	BIV	B	LC430561
ZAM-16-2017	B	BIV	NS	NS
ZAM-17-2017	A	AII	A	LC430562
ZAM-18-2017	B	BIV	B	LC430563
ZAM-19-2017	A	AII	A	LC430564
ZAM-20-2017	B	BIII/BIV	B	LC430565
ZAM-21-2017	B	BIV	B	LC430566
ZAM-22-2017	B	BIII/BIV	B	LC430567
ZAM-23-2017	A	AII	A	LC430568
ZAM-24-2017	B	BIV	B	LC430569
ZAM-25-2017	B	BIV	NS	NS
ZAM-26-2017	A	AII	A	LC430570
ZAM-27-2017	B	BIV	B	LC430571
ZAM-28-2017	B	BIV	B	LC430572
ZAM-29-2017	A	AII	A	LC430573
ZAM-30-2017	A	AII	A	LC430574
ZAM-31-2017	B	BIII	B	LC430575
ZAM-32-2017	B	BIV	B	LC430576
ZAM-33-2017	B	BIII	B	LC430577

NS - Not sequenced.

### 3.2 Phylogenetic analysis of gdh gene

**Fig. 1:**
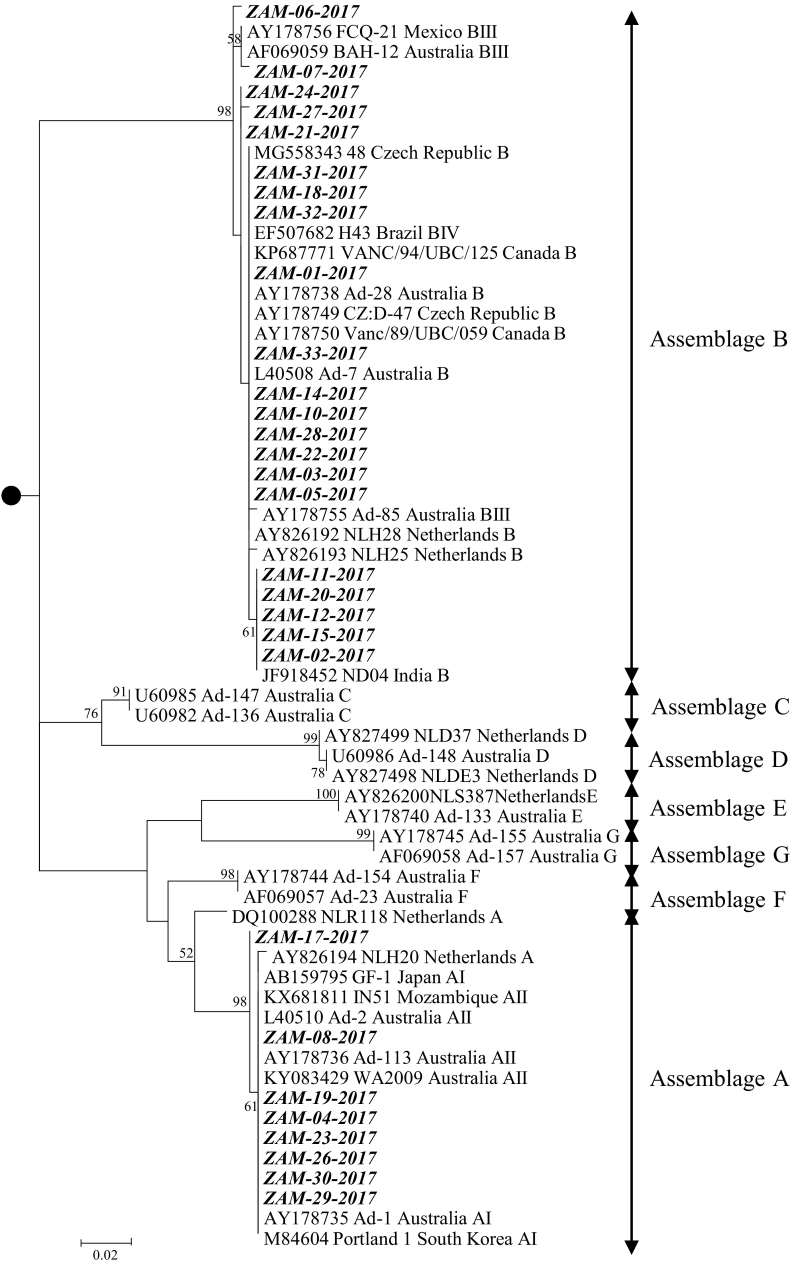
Phylogenetic analysis of *G. duodenalis* detected from human fecal samples in Zambia. The analysis was based on the *gdh* gene and involved 63 nucleotide sequences, with a total of 318 positions in the final dataset. The phylogenetic tree was rooted (black circle) to *G. ardeae* (GenBank accession number AF069060). The numbers at branch nodes represent bootstrap values ≥50%. Reference sequences included in the analysis are shown with their respective GenBank accession numbers, strain name, country of origin and genotype. *G. duodenalis* strains characterized in this study are shown in bold italic text. Bar, number of substitutions per site.

From the 33 DNA samples, 29 (87.9%) isolates were successfully sequenced (Table 1). At the nucleotide level, the *gdh* gene sequences of *G. duodenalis* isolates detected in Zambia shared 89.8% to 100% similarity whilst the predicted amino acid sequences showed 97.2% to 100% sequence identity. By the Basic Local Alignment Search Tool (BLAST) (https://blast.ncbi.nlm.nih.gov/Blast.cgi) analysis, the nucleotide sequences of the *gdh* gene of *G. duodenalis* of Zambian assemblage A (as determined by RFLP analysis) strains were highly similar (99–100%) to isolate Ad-2 (accession no. L40510) isolated from humans in Australia (Monis et al., 1996). Also, Zambian assemblage B *gdh* sequences were highly similar (99–100%) to isolate NLH25 (accession no. AY826193) found in humans in the Netherlands (van der Giessen et al., 2006). Phylogenetically, the *gdh* gene sequences of *G. duodenalis* were separated into various assemblages (Fig. 1). Phylogenetic analysis of isolates examined in this study revealed that 27.6% (8/29) clustered with assemblage A isolates, whilst 72.4% (21/29) belonged to assemblage B (Fig. 1).

## 4 Discussion

In the present study, we report a prevalence of 10% (33/329) in the sampled population, which is lower when compared to previously reported studies in Zambia. Contrary to the study by Siwila et al. (2010) which focused on children living in a peri-urban area and that by Graczyk et al. (2005) which focused on children in a rural setting, our study focused on children in an urban area and this could explain the differences in prevalence reported. Urban settings tend to have better infrastructural development than in peri-urban and rural settings. Indeed, the level of development of a community and its education (especially the level of education of mothers) has been reported to be risk factors in the epidemiology of giardiasis (Al-Mekhlafi, 2017; Karan et al., 2012). The higher prevalence observed by Graczyk et al. (2005) could also be due to the fact that they sampled children who were exhibiting diarrheal symptoms, contrary to those from our study, considering that *Giardia* is considered to be a common cause of diarrhea (Torgerson et al., 2015). It is also possible that the prevalence reported in our study is lower than those reported by Siwila et al. (2010) and Graczyk et al. (2005) due to the difference in techniques used. Our study used simple microscopy whilst the other two studies used the more sensitive fluorescence microscopy, thus our study could have under-reported the prevalence. The overall prevalence in our study could also have been underestimated because we used a single stool sample which has been reported to have a significantly low sensitivity (46%) when compared to the recommended 3 stool samples over 3–5 days (94% sensitivity).

In this study, sampling was conducted only during the dry season and this could have resulted in an underestimation of the overall prevalence of infection in this population as it has previously been reported that there is a positive correlation between wet weather conditions and increased prevalence of Giardia infection in populations (Britton et al., 2010; Siwila et al., 2011; Lai et al., 2013). It is postulated that the increased runoff during rainy season leads to increased contamination of water bodies, with consequent increased transmission of pathogens to humans (Galway et al., 2015). Also, the increased turbidity of water during rainy season, due to runoff, reduces the effectiveness of water treatment using chlorine (Schwartz et al., 2000), leading to increased pathogen transmission. Considering that our study site is characterized by inadequate sanitation and water supply, resulting in many households relying on shallow boreholes for drinking water, it is possible that there could be increased transmission in the rainy season than the dry season. Therefore, it is important to conduct a similar study covering the wet season in order to better understand the epidemiology of Giardiasis in this community.

The finding of *Giardia* in non-diarrheal children in our study further adds to observations by other authors that *Giardia* could be a commensal in children from high prevalence settings (Bartelt and Platts-Mills, 2016). Indeed, some studies have reported comparable prevalence in both symptomatic and asymptomatic children (Anim-Baidoo et al., 2016; Jerez Puebla et al., 2017). Such asymptomatic cases are important as possible sources of infection for people in close proximity and pose a risk of infecting other children in areas such as schools (Oliveira-Arbex et al., 2016).

Whilst some studies (Damitie et al., 2018; Julio et al., 2012) have reported that younger children have higher prevalence of infection with *Giardia*, this was not the case in our study. In fact, though not statistically significant, we observed an increase in prevalence with increased age. This finding of increased prevalence with increased age has also been reported in other studies (Al-Mekhlafi et al., 2013; Anim-Baidoo et al., 2016).

Contrary to the findings by Siwila et al. (2010) that sex/gender was a significant risk factor for *Giardia* infection with more girls infected than boys, our study did not find a significant difference in infection rates between sexes. Our findings are in agreement with those of other authors (Damitie et al., 2018; Julio et al., 2012) who found no association between sex and *Giardia* infection. It is possible that the effect of sex on infection could be context-specific and is not a general phenomenon, given the variation in reports.

Considering that most community schools lack the basic amenities, it would be expected that children attending these schools would have higher infection levels than those attending public schools which have better amenities. This is because lack of basic sanitary conditions is one of the main risk factors for *Giardia* infection. It was therefore surprising that in our study, children attending community schools had significantly lower infection levels than those from public schools. The observed high prevalence in children from public schools could be explained by the high enrolment levels in these schools, which would put a burden on the available amenities. This would possibly increase contact, reduce hygiene in the school environment and enhance transmission. As such, construction of more amenities at public schools could help reduce the transmission. Alternatively, construction of more schools, so as to reduce the student population per school, would result in reduced contact between children, ease the burden on amenities and improve hygiene at schools thus consequently reduce disease transmission.

Phylogenetic analysis, based on the *gdh* gene, showed that our isolates clustered with isolates of assemblages A and B (Fig. 1) and this is not surprising considering that these assemblages are well-known to infect humans (Caccio et al., 2017). There was a predomination of assemblage B in our study (73%) and this is in agreement with most studies conducted across the world (Ryan and Caccio, 2013). In comparison to other assemblages that have been reported to be infective to humans, assemblage B transmission has mainly been associated with human hosts, who serve as a source of infection (Sprong et al., 2009) and this possibly explains its predominance in areas of limited hygiene and sanitation. In our study, we did not find any statistically significant association between infecting *Giardia* assemblage and either of sex or age group, a finding which is in agreement with other studies (Jerez Puebla et al., 2017; Rostaminia et al., 2017).

Further characterization of the isolates in our study based on RFLP of the *gdh* gene showed the presence of sub-assemblages AII, BIII and BIV. Finding of only sub-assemblage AII was not surprising considering that within assemblage A, this particular sub-assemblage (AII) has mostly been identified in humans whereas sub-assemblage AI has been found mostly in animals (Caccio and Ryan, 2008). We also found sub-assemblages BIII and BIV, which are also normally found in humans (Monis et al., 2003), with some mixed infections of sub-assemblages BIII and BIV. Contrary to findings by Sprong et al. (2009) that sub-assemblage BIII was more prevalent in Africa, sub-assemblage BIV was more prevalent in our study. Predominance of sub-assemblage BIV in our study is in agreement with findings from studies in Ethiopia (de Lucio et al., 2016), Kenya (Mbae et al., 2016) and Tanzania (Di Cristanziano et al., 2014). Assemblage and sub-assemblage co-infections have been reported before in other studies (Faria et al., 2016; Gelanew et al., 2007; Hussein et al., 2017; Mbae et al., 2016), and their occurrence has been hypothesized to be due to repeated and cumulative infection in an area (Roointan et al., 2013) as well as human exposure to multiple sources of infection (Damitie et al., 2018).

Whilst multilocus typing is recommended for genetic characterization (Broglia et al., 2013), single locus typing using the *gdh* gene has been widely used (Reviewed by Caccio and Ryan, 2008). The advantage of multilocus typing is that it increases the chances of successful PCR amplification as there may be nucleotide mismatches between the PCR primers and the genomic sequences, which may result in non-amplification of some isolates (Broglia et al., 2013). However, in our study, the *gdh* gene of all the microscopically positive samples was successfully amplified.

In conclusion, to our knowledge, this is the first report of genotypic characterization of *G. duodenalis* circulating in Zambia, revealing the circulation of assemblages A and B in school-going children in Lusaka District. The predominance of assemblages AII, BIII and BIV, which are mostly associated with human infections, suggests that anthroponotic transmission plays a major role in the epidemiology of giardiasis within the studied community. As such, control measures aimed at improving hygiene in this community, along with education campaigns could go a long way in curbing giardiasis.

## Declaration of competing interest

All the authors declare that there is no conflict of interest.
